# *Changing the Child*: The Impact of Novel Treatments on the Identity of Children With Monogenic Neurodevelopmental Disorders

**DOI:** 10.1111/bioe.70119

**Published:** 2026-05-15

**Authors:** Sietske A. L. van Till, Maartje H. N. Schermer, Hilgo Bruining, Matthijs Verhage, Eline M. Bunnik

**Affiliations:** ^1^ Department of Public Health, Program for Medical Ethics, Philosophy and History of Medicine Erasmus MC, University Medical Centre Rotterdam Rotterdam the Netherlands; ^2^ Erasmus School of Health Policy & Management Erasmus University Rotterdam Rotterdam the Netherlands; ^3^ N=You Neurodevelopmental Precision Center, Amsterdam Neuroscience, Amsterdam Reproduction and Development Amsterdam UMC Amsterdam the Netherlands; ^4^ Department of Child and Adolescent Psychiatry Amsterdam UMC, University of Amsterdam Amsterdam the Netherlands; ^5^ Levvel, Center for Child and Adolescent Psychiatry Amsterdam the Netherlands; ^6^ Child and Adolescent Psychiatry and Psychosocial Care Emma Children's Hospital, Vrije Universiteit Amsterdam, Amsterdam UMC Amsterdam the Netherlands; ^7^ Department of Functional Genomics Center for Neurogenomics and Cognitive Research (CNCR), Vrije Universiteit Amsterdam and Amsterdam UMC Amsterdam the Netherlands; ^8^ Department of Human Genetics, Center for Neurogenomics and Cognitive Research (CNCR) Vrije Universiteit Amsterdam and Amsterdam UMC Amsterdam the Netherlands

**Keywords:** authenticity, identity, neurodevelopmental disorders/genetics, pediatrics, treatment development

## Abstract

Scientific advancements offer new possibilities for developing novel treatments for children with monogenic neurodevelopmental disorders (mNDDs), targeting the underlying effects of their condition. Although, in qualitative studies, parents of children with mNDDs have predominantly positive attitudes towards the development of novel treatments, a few parents express concerns about whether treatments could change *who their child is*. Existing literature on personal identity, including literature on how personal identity is affected by medical (neuro‐)interventions, can assist in clarifying these concerns. This paper provides an analysis of interview data on perspectives of parents with children with mNDDs (STXBP1‐Related Disorders and Kleefstra Syndrome) on the impact of novel treatments on their child's identity. We identify four sets of concerns, about whether treatments (1) enable children to develop their self, (2) alienate children from their true self, (3) disrupt children's relational identities, and (4) defy children's horizontal identities. While parents hope that treatments will help children flourish, some worry that treatments may alienate children from their self or alter children's personal characteristics which are meaningful to caregivers, thereby disrupting family dynamics. Others fear treatments may produce stigma by perpetuating the narrative that children need to be “fixed”. This analysis helps to understand the ambivalence some parents express when considering their child's participation in clinical trials. For clinicians and researchers, it is essential to discuss parents' concerns about impacts of treatments on their child's identity (if present) when deciding on the child's participation in clinical trials, and to monitor children for identity‐related effects of novel treatments.

## 1 Introduction

Monogenic neurodevelopmental disorders (mNDDs), caused by de novo single‐gene variations, manifest in a heterogenous spectrum of symptoms [1]. While some children are mildly affected, many exhibit severe phenotypes, including seizures, cognitive impairments, autism‐like features, incontinence, and inability to walk, talk, or eat [2]. When young children are diagnosed, it is often unpredictable how their development will progress over time, and whether it may deteriorate during/after puberty (a phenomenon called regression) [3]. Current care practices are limited to symptom management, but scientific advancements offer possibilities for developing a new generation of medical treatments. Various research projects are currently investigating medicinal approaches to restoring neuronal function in mNDDs [Textbox 1] [4, 5, 6, 7]. For example, in the Dutch BRAINmodel project, neuronal cell models are used to identify *disease‐modifying* treatments, which target underlying mechanisms of mNDDs. While most approaches are in early stages of clinical development, they represent promising endeavors in what might be a paradigm shift in the medical treatment of mNDDs. Yet, these approaches pose questions about how novel treatments may impact children's wellbeing and their developing identities.

Textbox 1: **Types of medical treatment**

This study focuses on parental concerns about novel *disease‐modifying* treatments, but should be considered in the broader context of the development and implementation of a new generation of novel mNDD treatments. In the field of NDDs, of which mNDDs are a subset, treatments are classified into *symptomatic* treatments, *disease‐modifying* treatments, and *disease‐altering* treatments. These categories reflect distinct therapeutic strategies, which (presumably) have distinct clinical effects.

*Symptomatic* treatments are used to alleviate specific symptoms associated with NDDs, without targeting their underlying mechanisms. These treatments include conventional treatments like Ritalin/Adderall, sleep medication, antipsychotics, and antiepileptic drugs, which are broadly used in NDD patient groups. These treatments are limited by their nonspecific effects, often leading to suboptimal efficacy and unwanted side effects.

*Disease‐modifying* treatments represent a more targeted approach, targeting specific underlying mechanisms of mNDDs, typically at the cell or protein level. For instance, Bumetanide, a diuretic that modulates chloride ion transport in neurons, is being used to treat epilepsy and autism. The Dutch BRAINmodel project uses patient‐derived stem cell‐based neuronal cell models, in combination with EEG data and clinical phenotype, to identify *disease‐modifying* treatments for individual children with mNDDs. Still, children undergoing these treatments are unlikely to experience complete recovery, and any improvements remain inherently partial rather than curative.

*Disease‐altering* treatments form a relatively new class of therapies that target the genetic cause of mNDDs, using DNA‐ and RNA‐based technologies like antisense oligonucleotide (ASO) therapies and gene (replacement) therapies to directly target the primary deficit, the causal genetic variant. These therapies hold promise for a more fundamental approach to treating NDDs by *altering* the genetic cause, but often require invasive procedures, such as lumbar punctures. *Disease‐altering* treatments are still in the early stages of development and their clinical success remains uncertain.

Insight in the perspectives and lived experiences of parents is fundamental when developing and implementing novel mNDD treatments, so that they can be offered in a way that they will benefit children and their families, and respond to their needs. As primary decision‐makers and advocates for their children, parents also play a crucial role in deciding what kind of treatments they want for their child and under what conditions. In an interview study we conducted among Dutch parents of children with rare mNDDs (STXBP1‐Related Disorders and Kleefstra Syndrome, *n* = 28), we showed that parents had predominantly positive attitudes toward *disease‐modifying* treatments, and hoped that these treatments will improve children's wellbeing [8, 9]. However, when asked about their perspectives on the participation of their child in future clinical trials of disease‐*modifying* treatments, some expressed concerns. They were worried about medical risks of treatments, deteriorating effects on their child's development, and side effects, like epileptic seizures, somatic problems, or psychiatric decompensations. Interestingly, some parents mentioned another type of concern: they deemed the preservation of “who their child is” important, stating that they might decide against enrolling their child in clinical trials, because he/she “just is who he/she is”.

Some respondents expressed concerns related to prior experiences with conventional, *symptomatic* medical treatments. For instance, two respondents reported that anti‐epileptic treatments had previously made their child numb and seemingly less aware of itself and its environment, and thus less able to be “who he/she was”. These respondents had switched or discontinued their child's treatment, despite potential negative consequences for seizure management. Other respondents stressed that they were familiarized with who their child was, and struggled to envision how novel treatments might change their child. Some viewed the child's mNDD as an integral part of their identity, which should not or cannot be treated. A Canadian questionnaire study among parents of children with Down Syndrome (*n* = 101) reported similar concerns that “cures” might change children's personalities [10]. Turbitt et al. highlighted the potential impact of novel treatments on the identity and authenticity of children with genetic NDDs as a particular—yet unexplored—ethical concern [11]. To enable clinicians and researchers to address this concern, in research priority‐setting, research communication, and informed consent, it must first be clarified.

Concerns about impacts of medical treatments on a person's identity are not new. In disorders of the brain, it is not always clear to what extent “who someone is” is affected by the disorder [12, 13]. Medical treatments targeting psychiatric or neurological disorders may affect cognitive, psychological, communicative, and behavioral traits that are considered constitutive for who someone is. These treatments may be seen either as making disorder‐related traits less dominant or disappear altogether, thereby revealing who someone is, unhindered by their disorder, or as obscuring traits that are perceived as fundamentally linked to the person, thereby making part of them disappear. This tension has been described in cases like antidepressants, or Deep Brain Stimulation (DBS) in neurological patients [12, 13, 14, 15, 16, 17]. The existing literature on identity, however, primarily focuses on autonomous adults or patients who were autonomous before developing disorders like dementia, depression, or psychosis. Introducing novel treatments for children with mNDDs brings new challenges. Children's identities are still in development, and many children have cognitive impairments or speech difficulties that limit their ability to articulate their sense of self or become autonomous, even as they grow older. Consequently, these children's identities are often perceived through the lens of their caregivers. These distinctive characteristics add complexities in the attempt to understand how mNDDs affect children's identities, and consequently, how treatments may impact these identities when targeting mechanisms underlying mNDDs.

This article aims to provide a theoretically and empirically informed understanding of parents' concerns by relating philosophical accounts of identity to interview data. In this way, we provide a language to better articulate these—often implicit—concerns. First we distinguish four sets of concerns. Afterwards, we discuss how our analysis can help understand concerns about impacts of medical treatments on children's identities, and the implications for development and implementation of novel mNDD treatments.

## 2 Methods

This study builds on our interview study investigating parents' lived experiences of caring for children with mNDDs and their perspectives on the development and implementation of *disease‐modifying* treatments for mNDDs. Methods and main results of this study are published in separate articles [8, 9]. Parents of 28 children with Kleefstra Syndrome or STXBP1‐Related Disorders (ages 2–25) were included. They received information about *disease‐modifying* treatments through an informational video, written information, and oral explanation by the interviewer. As *disease‐modifying* treatments are not yet broadly used in clinical practice, parents had no experience with these treatments. Therefore, their perspectives were often informed by prior experiences of symptomatic treatments.

For this study, we performed a secondary analysis of interview data to identify fragments expressing parents' concerns relating to children's identities. Additionally, we conducted a literature study on philosophical accounts of personal identity, including literature on experiences‐of‐self of individuals with neurological and psychiatric disorders, and on identity in the context of cognitive enhancement and psychiatric/neurological interventions. In our interview data, we identified four main sets of concerns about impacts of novel treatments on children's identities. We used existing literature on identity to help understand the concerns of parents who are considering novel treatments for children with rare mNDDs. We use quotes by parents to illustrate these sets of concerns.

## 3 Results

We identified four sets of parental concerns about the impact of novel treatments on children's identity (Table 1). We discuss these concerns in relation to four theoretical accounts, featuring different dimensions of identity.

**Table 1:** Four sets of parental concerns about the impact of novel mNDD treatments on children's identity.

Treatments could potentially change the child by…	Dimensions of identity	Concern
Enabling the child to develop their self	*Who the child is* in relation to their future self	The child has the right to flourish
Alienating the child from their true self	*Who the child is* in relation to their essence	It could be harmful for the child to be alienated from itself
Disrupting the relational identities of the child	*Who the child is* in relation to their close social environment	If the child changes, the family as a whole will change, as well
Defying the horizontal identities of the child	*Who the child is* in relation to their social group	The child should be accepted *as it is* by society

### 3.1 Enabling the Child to Develop Their Self

Parents are interested in novel treatments because these treatments may strengthen their child's ability to develop their self—or even their “authentic” self. Although there are differing accounts of “authenticity”, being one's authentic self is generally taken to mean something like living *in accordance with one's* “true self,” which is considered instrumentally important for wellbeing and autonomy, and, according to some views, also has intrinsic value [12, 17, 18, 19]. The first set of concerns about whether a treatment may induce changes in the child's identity, relate to existentialist accounts of authenticity. Existentialist accounts of authenticity generally imply that becoming one's self involves a process of self‐development or self‐creation, which allows, or requires, changes in personal characteristics [12, 15, 17]. According to existentialist accounts, the self is not fixed at birth, but actively created and chosen by persons themselves. Pathological conditions, such as neurological and psychiatric conditions, may hinder the process of self‐development/self‐creation and should thus be treated [15, 17, 20, 21]. For children, whose identities are still developing, novel treatments may support their “normal” development, and enable them to become their self.

Cognitive capacities like imagination, memory, and introspection are important for self‐creation, because persons need to make autonomous choices about who they want to be [22]. However, arguably, self‐development already begins before children develop such capacities. The first steps in self‐development are often intuitive (children conduct actions non‐deliberately that ultimately define who they are) and steered by caregivers. This early process of self‐development is important for children's right to have an open future. Parents must support children's development, so that, as children turn into adults, they become capable of making autonomous choices about who they want to be [23]. For self‐development, a broad range of capabilities needs to be developed during childhood. The capability approach, for instance, stresses the importance of certain capabilities (e.g., speech and language, engaging in recreational activities, movement) for achieving wellbeing and enabling self‐development. These capabilities are necessary to have valuable opportunities, including being educated, being healthy, being part of a social network, or getting married [24].

MNDD treatments may be a means to support children in self‐development. In a “normal” development, children acquire skills and abilities within a typical timeframe. In children with mNDDs, pathogenic genetic variations hinder this. Though clinical phenotypes may vary widely, many children with mNDDs do not develop at least some capacities required for self‐development. First, some lack the cognitive capacities to consciously reflect and make decisions about who they want to be, also when they grow older. Second, capacities such as speech, mobility, and sensory processing may be underdeveloped, cutting off possibilities in their self‐development. Medical treatments can foster children's development and, thereby, expand their opportunities to construct or evolve their (true) self, and to flourish.

In our interview study, parents described tremendously limiting impacts of children's genetic conditions on who they currently are, and who they will be able to be in the future. For instance, parents worried that their child will not be able to interact with peers, live independently, get an educational degree, have a job, or build a family. Some parents felt that under these constraints, their child could not be their self. A mother was enthusiastic about the development of novel treatments…:

“Because I believe that that boy is a very lively boy who could have led a completely different life than the one he is forced to live now.”

Some parents hoped that novel treatments could “repair” or “fix” the genetic condition, enabling their child to become “normal”. However, they acknowledged that *disease‐modifying* treatments will probably not reverse all mNDD symptoms. Still, they hoped that treatments could induce small improvements, like enabling their child to say some words, read, go to the toilet independently, make sense of situations, walk, or regulate behavior more effectively. These improvements would increase children's independence and put them more *in control*. But above all, they would allow children to develop their own—authentic—selves.

### 3.2 Alienating the Child From Their True Self

While novel mNDD treatments may cause improvements in some capacities, questions arise about whether these improvements may, in fact, alienate the person from their “authentic” self. This second set of concerns relates to essentialist accounts of authenticity. In contrast to existentialist accounts, essentialist accounts hold that a person's core characteristics are pervasive, biological, and consistent [12, 25, 26].

Differing views exist on what a person's “essence” encompasses: in some views, a person's essence corresponds to their “healthy” essence: who they would be without their disorder. Here, pathological conditions, such cognitive impairments, may be seen as exogenous factors, as artifacts hindering the person to engage in a process of introspection, and thereby, to discover and consciously endorse their true self [12, 14, 15, 27]. In other views, a person's essence corresponds to the person as they are, as a “natural self” with all their innate characteristics, including the disorder causing developmental delays. Following these views, some argue that alterations to the “natural” or “given” self, induced by interventions like psychotropic drugs or gene therapies, are undesirable [12, 28]. Here, being authentic means being faithful to one's “natural” self. Even if interventions improve a person's personality characteristics (e.g., make them more empathetic), they are not seen as improvements of the person as a whole: these “improved” characteristics are no longer theirs [26, p. 29]. In the bioethical literature, the notion of a “natural” or “fixed” essence is frequently problematized [29, pp. 232–242]. Still, some parents in our interview study refer to the mNDD as being an integral part of their child: the child is simply who it is, having no other option than being its “pure” self with their unique genetically predisposed development, character, and behavior. The mNDD cannot be separated from the child, because it is constitutive of who they are. For instance, autistic self‐advocate Jim Sinclair argues that being autistic “colors every experience, every sensation, perception, thought, emotion, and encounter, every aspect of existence”: it is essentially a different way of being [30]. Sinclair would argue that if treatments would cure a child's autism, the child would be a fundamentally different child.

Some argue that if medical treatments induce changes to self‐constitutive core characteristics, or provoke side effects like emotional or behavioral dysregulation, they may cause experiences of self‐alienation in the affected patient [14, 15, 22]. *Alienation* (“being separated from what is most their own”) is discussed in relation to treatments like Ritalin for ADHD, antidepressants, and DBS for neurological disorders [21, 31, 32]. Children with mNDDs will likely not understand the implications of novel treatments nor be able to decide for themselves whether they want to use them. This might increase the risk that, after treatment, children feel like a different person, experiencing traits that are unrecognizable to them. Parents of children with Down syndrome were found to have concerns about whether their children would have to “relearn” who they are after being “cured” [10]. During our interviews, parents expressed fear that children might become frustrated or depressed because, while they may be (partly) “treated”, they may feel uncomfortable in their newly acquired existence. A mother explained:

“maybe he would end up being able to talk [after treatment], but then I wonder, what am I really doing to him? And it [his condition] is such a complex thing, because he is ‘in balance’ now. Everything about him just fits together as he is. If one part were to improve, would I end up with a frustrated child? Because then he would not be in the balance he is meant to be in.”

Parents expressed concerns that children would become surrogates of who they are, even if novel treatments were to lead to some improvements in specific domains, such as seizure‐management or motor skills. For example, treatments could turn children into “zombies” or “plants”, or make them less aware of themselves or their environment. Some parents observed such effects in their own child or in other children after symptomatic treatments, like anti‐epileptic or behavior treatments. If children could no longer “be themselves” during/following treatment, these parents would consider to refuse or discontinue treatment.

### 3.3 Disrupting Children's Relational Identities

The third set of concerns covers children's *relational* identities, which develop in interconnectedness with particularly their families and might be disrupted through novel treatments. This dimension reflects that humans are *social* by nature [33].

The relational account of identity emphasizes the role of persons' bodily expression of needs, emotions, and thoughts, and how these are recognized by their environment [34]. Children with NDDs often have unique, sometimes non‐verbal, ways of communicating [35]. Over time, parents become attuned to their child and pay “much closer attention to—and honouring, by giving epistemic credence to—these non‐verbal forms of communication” [36]. Furthermore, parents contribute to and maintain their child's narrative identity. Even if children cannot “actively” contribute to their own narrative, those around them can shape the story of who they are and treat them accordingly. Lindemann described how her and her family's interactions with her sister who had severe cognitive impairments shaped her sister's identity: “Because I played with her, she was my playmate: Because my mother cared for her at home, she was a member of the household”. This *social practice of personhood* has both descriptive dimensions, through interpreting and articulating the child's identity, and normative dimensions, “by acknowledging a loved one's personhood with our own” [34].

Simultaneously, raising a child with an mNDD shapes family members' identities. While parental experiences differed, nearly all respondents reported that raising their child had significantly changed their lives [8, 9]. For instance, parents had to watch over their child, organize care and support, and divide their time between care responsibilities and other domains of life (work, caring for siblings, social activities), and entered a new world of caregiving. Moreover, parents had to cope emotionally with the reality of raising their child, instead of a previously expected “typical” child. Families adjust emotionally and practically to their child's capacities and needs, and create a balance, a “new normal,” which is often extreme but stable [37, p. 366].

Given the entanglement of children's and parents' identities, it is uncertain how mNDD treatments would change their relational identities and whom it affects most: children or parents. Lipsman and Glannon suggest that changes in personality characteristics through DBS may not necessarily disrupt the narrative that patients hold for *themselves* but rather the narratives *others* hold for them [20]. In our interviews, parents questioned how treatments might change their relationship with their child:

“That he's such a cheerful little boy now, I wouldn't want it any other way. […] I think I would find it really hard to imagine him suddenly being able to talk or maybe being able to learn something. Don't get me wrong, I'd love that, of course! That [child's name] could just play outside with friends here in the street. […] But I think that's also because I've become accustomed to the situation and have accepted it. That he is as he is, that he will never be ‘normal’[…].”

Another parent mentioned not wanting to lose their child *as he is* because he made a unique (and often positive) contribution to their family. Also, many parents provided vivid descriptions of their children, labeling them as being sweet, brave, or humorous. These descriptions portrayed their children's character and behavioral predispositions (essentialist account), but also showed the social practice of relational identity‐building at play. Concerns about whether treatments will “change” children are especially pertinent in treatments targeting behavior, mood, or speech, as these aspects are central to their relational identity.

Concerns about relational identity were mainly expressed by parents with older children. This is likely because relational identities are not fixed but rather take time and effort to establish and evolve. After this process, parents might struggle to imagine they would change *their* child into a hypothetical “better” child through treatments, and thereby adapt, themselves, to being a parent of this “better” child.

### 3.4 Defying Children's Horizontal Identities

The final set of concerns involves views of disability not as inherently bad, but as a form of diversity with *social* meaning. It focuses on the role of relationships and identification with certain communities, in this case the social group of individuals with mNDDs, as an important factor in defining these individuals' identities.

One way to conceptualize social identities is through the distinction between horizontal and vertical identity, as proposed by Andrew Solomon, who describes disability as an example of a horizontal identity [37]. Vertical identities are constituted through characteristics that are inherited from parents and passed down across generations, including genetically transmitted traits (e.g., eye colour, skin colour), as well as culturally transmitted characteristics like religion and language. A horizontal identity, by contrast, is shared with others within society, a peer group, with similar characteristics, and often similar experiences and interests. These shared characteristics, such as de novo genetic mutations, arise randomly in individuals, and are not shared with their parents. In this case, the disability affecting a child with an mNDD is not something relatable to their parents, but can be considered a horizontal identity, shared with those similarly afflicted.

Horizontal identities are not neutral but carry social meaning: they shape how individuals are perceived by themselves and others. Lindemann refers to this socially constructed identity as a *master narrative*: “familiar stories embodying our culture's socially shared understandings” [38, p. 71]. Many authors have criticized existing narratives about disability. For instance, Stramondo has shown how “disability” is colored by assumptions about disabled people being *needy, helpless, incompetent, or suffering* [39]. Importantly, negative assumptions regarding disability might simply be mistaken. Also, they have negative consequences for disabled persons. On a personal level, these narratives may hinder disabled people from having positive and fulfilling perspectives on their own identities, because these are in constant tension with dominant stereotypes of their disabilities [40, p. 173]. On a social level, these narratives are often stigmatizing and can ultimately lead to inadequate care and support for disabled individuals. Differing perspectives exist on what the master narrative of disability should entail. Barnes proposes a value‐neutral model of (physical) disability, arguing that being disabled makes someone part of a minority group, but is not a *way of being inherently or intrinsically worse off* [40, pp. 78–118]. Especially within the neurodiversity movement, debates continue about what NDDs are, and whether and how the wellbeing of people with NDDs should be improved.

The development of novel treatments may influence the master narrative of mNDDs. Attempts to “fix” conditions through medical treatments may contribute to stigmatization and pathologization, by perpetuating the narrative that affected individuals are “defective” [39, 41, 42, 43]. Additionally, it may contribute to the idea that affected individuals and their families should prioritize finding treatments to *overcome* and cure their condition, rather than finding a fulfilling way to live *with* their condition [40, p. 164].

In our interview study, some parents expressed reluctance toward using treatments for their child, because they believed that treatments serve the needs of others, but not their child. For example, some argued that treatments targeting “problematic” behavior aim not to benefit children but to have them conform to social norms. Some parents questioned their right to impose treatments on their child when he/she seems content and not in medical need:

“And what are we really giving her with this [treatment]? I think that's also an essential point. Not everything is fixable: she is who she is. And part of it lies in acceptance on our part. So if the child is like this and the child is happy with it, is it then up to me to change something about that?”

While some of these parents did not want novel treatments for just their own child, others saw treatment‐development as part of the broader discussion on how children with mNDDs, as a group, should be treated. These parents advocated for focusing on children's wellbeing and promoting social acceptance. In general, parents did approve of some therapies or assistive technologies (e.g., physiotherapy and speech computers), aimed at fostering children's development and social participation. Yet some felt that children should not be forced to use medical treatments aimed at making them “normal”, as this implies they are not well in their untreated state.

## 4 Discussion

Parents generally have positive attitudes toward the development and implementation of novel mNDD treatments, yet a few express concerns. While most concerns relate to medical risks, we highlight a relatively underexplored issue. Our analysis of interview data reveals four sets of concerns about potential impacts of novel treatments on the developing identities of children with mNDDs, and helps to understand the *ambivalence* some parents express when considering their child's participation in clinical trials. Parents often go back and forth between wanting to improve their child's wellbeing and flourishing, and wanting to protect them against possibly alienating effects of treatments. Parents tend to value aspects of their child in the present which they do not want them to lose. Additionally, some fear that drug development efforts for mNDDs may provoke stigmatizing narratives about children with mNDDs.

In our analysis, we employed several accounts of personal identity. Concerns expressed by parents particularly connect to a qualitative understanding of personal identity, often labeled as *narrative identity*, as distinct from *numerical identity* [29]. Numerical identity is typically used to address whether an individual remains the same person over time. In our study, parents did not question that their child would still be their child in a numerical sense. Instead, their concerns seem to connect more to discussions on novel treatments potentially causing biographical disruptions in their child's *narrative identity*: the story of who this person is, incorporating the person's core characteristics such as their actions, psychological traits, values, roles, and social relationships [29, 44].

Within this narrative conception of personal identity, we identified three axes along which parents' concerns can be mapped.

First, our findings resonate with ongoing debates on whether current psychiatric and neurological treatments might reveal or obscure a person's authentic self [12, 13, 15, 17]: according to some essentialist accounts, being authentic means to stay true to one's essence, embracing—and not rejecting—one's innate predispositions, which determine who one is. In contrast, according to existentialist accounts, treatments may help children overcome the symptoms associated with their condition, and gain the capability to actively create their “authentic” self.

Second, we show that the narrative of “who the child is” can be approached from both the first‐person and third‐person perspective. Traditionally, narrative identity is often tied to the person's self‐conception, thereby privileging the first‐person perspective [29, pp. 83–88]. Accordingly, existing literature on impacts of treatments on personal identity often focuses on potential impacts on a person's self‐conception, such as first‐hand experiences of self‐alienation, or of feeling (more) like becoming their authentic self by overcoming their physical or mental limitations [12, 15, 32]. However, we show that relational and horizontal accounts of identity are also at stake, accounts of identity that allow it to be construed and maintained from a third‐person perspective. Here, identity is (at least partly) narratively constructed and ascribed to a person by others, such as their family members and society at large [38]. This is especially relevant in the case of individuals with cognitive impairments, as their self‐conception is often unknown. “who they are” is mostly interpreted and articulated by others.

Third, existing literature on personal identity primarily reflects individualistic notions of the self, perceiving the self either as an essence lying within the individual (essentialism) or as being actively developed by the (autonomous) person themselves (existentialism). According to relational and horizontal accounts, a person's identity is (partly) shaped in relation to their social context, for instance, through relationships with their close family members and their membership of social groups with shared narratively‐constructed identities [37, 38]. Identity does not exist in a vacuum, especially not in children with mNDDs. Children's identities develop in interconnection with their caregivers, as they generally require continuous care and support, and, as all members of society, they share group‐identities.

In short, our study contributes to the literature by demonstrating that the conceptions of identity as developed in the literature are useful in better understanding identity‐related concerns of parents arising in the context of treatments for children with mNDDs. Still, these conceptions are also underdeveloped in some respects. While socio‐relational dimensions of identity have been explored within the discourse of care ethics, they are rarely applied or scrutinized within the literature on the impacts of neurological and psychiatric treatments on the personal identity of affected individuals [38]. Further conceptualization of the socio‐relational dimensions of personal identity is needed, for instance, to better understand the role of others in the construction of the child's identity (particularly in the absence of access to their first‐person perspective), the influence of the sometimes symbiotic relationships between parents and children on their mutual identities, and the interaction between the child's self‐conception and the child's ascribed identity.

### 4.1 Implications for Future Research

Now that we have identified four distinct sets of identity‐related concerns, we present a framework to support parents, researchers, and clinicians in further exploring and discussing these concerns during the decision‐making process about children's participation in clinical trials (see Table 1). We hope that this framework can provide parents with a language to better articulate their concerns. As such, our work can be understood as an attempt at improving hermeneutical justice for this group [45].

This study underscores the importance of addressing concerns beyond medical risks. As said, concerns about identity are not unique to newly developed mNDD treatments, and are also mentioned in relation to existing symptomatic treatments, such as anti‐epileptic therapies. Moreover, even when parents express concerns, they often do not oppose treatments outright, as they believe that treatments could also greatly benefit their child's wellbeing and development. Still, understanding these concerns can help researchers and clinicians address them more effectively.

Existing literature on identity related to medical conditions often presents a dichotomy: either a patient's condition is seen as integral to their identity and should be accepted (essentialism), or the patient's condition is seen as preventing the patient from creating their own self, requiring (medical) intervention (existentialism). However, this dichotomy risks either *pathologizing* children with mNDDs by labeling them as “defective,” or wrongly *depathologizing* them by insufficiently acknowledging the disorder's impact and severity—and providing insufficient care and support [46]. Relational and horizontal accounts of identity offer dynamic understandings of children's identity, recognizing, first, that how children's condition contributes to their identity may vary across families with their unique family dynamics. Second, the master narrative on mNDDs is not fixed, but shaped by society and, ideally, by advocacy organizations. So, to support families effectively, it is essential to move beyond the essentialist/existentialist dichotomy and to acknowledge the pluriform perspectives on identity among the mNDD community. For instance, some mNDD advocacy organizations, such as the *STXBP1* Foundation and Idefine (Kleefstra Syndrome), embrace broad approaches: they advocate for developing medical treatments targeting the disorder, and simultaneously for creating social awareness and enhancing social support services, helping to accept and cope with the disorder [47, 48].

Finally, the mechanisms and effects of novel treatments remain under investigation. While *disease‐modifying* treatments are not expected to effectively ameliorate all symptoms, scientists and clinicians hope that *disease‐altering* treatments will have more profound impacts, as they directly target *genetic* causes of the condition (see Textbox 1). However, *disease*‐*altering* treatments are potentially more disruptive and definitely more uncertain because to date so few have been tested in patients. Parents are worried that treatments may have unexpected effects on their child's sense of self, which are, indeed, difficult to foresee in this early stage of development. For example, research indicates that after surgery, epilepsy patients experience psychosocial and affective changes, and some, surprisingly, struggle to adjust to the psychological experience of being seizure‐free [49]. Moreover, anticipating the impact of novel mNDD treatments on children is particularly challenging because their identities are still developing. Being treated (or not) may steer their developmental trajectory by fostering the development of different capacities, values, and senses of self [50, p. 148]. For example, children growing up deaf might endorse different aspects of “who they are”, like having unique sensory experiences and being embedded in Deaf culture, than those growing up with cochlear implants [40, p. 57].

To better understand potential impacts of novel treatments, it is crucial to not only investigate medical risks in clinical trials but also identity‐related impacts, such as impacts on the social interactions between children and their family members, their mental wellbeing (e.g., expressions of frustration, confusion, or depression), and the development of capabilities that expand their range of opportunities. Additionally, impacts on children's horizontal identities cannot be assessed at the level of the individual child. Therefore, further research is needed on how novel treatments may influence socially shared understandings of mNDDs.

## 5 Conclusions

Scientific advancements hold great potential for introducing a new generation of treatments for children with mNDDs. This study investigates parental concerns about the impacts of novel treatments on children's identity by relating philosophical accounts to empirical data. Our analysis shows concerns about whether these treatments will help children flourish by targeting the hindering effects of their condition (existentialist account), or may introduce alienating traits (essentialist account). Moreover, concerns exist about whether treatments could disrupt relational identities by altering aspects of children that are recognizable to their parents or contribute to parents' own identities. Finally, the development of treatments poses concerns about whether they provoke stigmatizing narratives surrounding children with mNDDs, affecting their horizontal identities. For clinicians and researchers, it is essential to discuss these concerns with parents when deciding on the child's participation in clinical trials and to monitor children for identity‐related effects of novel treatments. We hope this study will provide the necessary language to do this.

## Funding

This manuscript is a result of the research project “BRAINMODEL: standardized, IPSC‐based medicine for immediate application in monogenic neurodevelopmental disorders”, which was funded by ZonMw—The Netherlands Organisation for Health Research and Development, project number 10250022110003 (2021–2027). ZonMw also provided a travel grant to support the literature study, which is conducted during an academic visit to the Uehiro Oxford Institute, project number 10250072310003 (2024–2025).

## Ethics Statement

This article builds on an interview study which is conducted in the context of the BRAINmodel project. For this interview study, we received a waiver from the research ethics review committee of Erasmus MC (MEC‐2022‐0155) on the 21rd of March 2022, as this type of study does not fall under the scope of the Medical Research Involving Human Subjects Research Act (WMO). Respondents gave verbal consent for participation in the study and for publication of the results, which was recorded on audio‐tape. For interview studies in the Netherlands, written consent is neither customary nor required.

## Conflicts of Interest

All authors declare that no support from any other organization is involved in the submitted work; no financial relationships with any organizations that have an interest in the submitted work; no other relationships or activities that could have influenced the submitted work.

## Transparency Statement

The lead author (S.T.) affirms that the manuscript is an honest, accurate, and transparent account of the study being reported.

## Data Availability

The authors have nothing to report.
